# Paternal Malnutrition has Organ‐Specific Intergenerational Effects on Mitochondrial Function and Oxidative Stress Induced DNA Damage in Male Mouse Offspring

**DOI:** 10.1002/mnfr.70470

**Published:** 2026-04-17

**Authors:** Esther S. Lenssen, Sonia de Assis, Alex Remels, Raquel Santana da Cruz, Frederik‐Jan van Schooten, Roger W. L. Godschalk

**Affiliations:** ^1^ Institute of Nutrition and Translational Research in Metabolism (NUTRIM) Maastricht University Maastricht, Netherlands The Netherlands; ^2^ Department of Oncology Lombardi Comprehensive Cancer Center Georgetown University Washington DC USA

**Keywords:** DNA damage, mitochondria, offspring, oxidative stress, Paternal diet

## Abstract

Paternal pre‐conceptional lifestyle may affect the offspring's levels of oxidative DNA damage. Therefore, we investigated whether paternal malnutrition affected mitochondrial function, oxidative stress, and DNA damage in lung and liver of mouse offspring at adult age. Adult male C57Bl6 mice received a control or low protein diet (LPD) and were mated with female mice reared on control diets. Offspring was kept under control feeding conditions until adult age. Compared to control, mitochondrial copy number and citrate synthase activity were higher in lung and liver of offspring whose fathers received LPD. Moreover, expression of genes involved in mitochondrial biogenesis was lower in lungs of offspring whose fathers received LPD, but no changes were observed in liver. Mitochondria may be a source of oxidative stress and indeed, levels of 8‐oxo‐deoxyguanosine (8‐oxo‐dG) were higher in lungs of offspring whose fathers received LPD. On the contrary, 8‐oxo‐dG levels in liver were lower in offspring whose father received LPD, which was associated with the tissue‐specific expression of enzymatic antioxidants catalase, NAD(P)H: quinone oxidoreductase 1 and γ‐glutamylcysteine synthetase. This study confirms that pre‐conceptional paternal malnutrition can influence background levels of DNA damage and mitochondrial function in a tissue‐specific manner in offspring.

## Graphical abstract

Preconceptional paternal exposure to a low protein diet influenced background levels of mitochondrial function, expression of antioxidant genes and oxidative stress induced DNA damage in a tissue‐specific manner in offspring. Paternal lifestyle might therefore explain part of the inter‐individual variation.

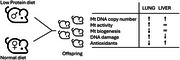

## 1 Introduction

Cells generate reactive oxygen species (ROS) as by‐products of metabolic and biochemical processes. Although, under normal physiological conditions, the production and scavenging of ROS are balanced, ROS may ‘escape’ from antioxidants and can damage macromolecules, including DNA [1]. As a result, 8‐oxo‐deoxyguanosine (8‐oxo‐dG), which is the most frequently studied pro‐mutagenic DNA lesion, can be detected in the DNA of healthy individuals and laboratory animals [2]. When the balance shifts to an overproduction of prooxidants, oxidative stress will result in the accumulation of DNA damage, which is a characteristic of many chronic degenerative diseases [2]. Even in healthy individuals, the levels of 8‐oxo‐dG can vary, and this inter‐individual variation cannot yet be fully explained. Although it is generally accepted that the levels of oxidative DNA damage are determined by the net effect of different processes, including the formation and scavenging of ROS, and the level of DNA repair [3, 4], researchers still struggle to explain the exact sources of inter‐individual variation [5], which may have a mixed environmental, genetic or epigenetic origin.

Various studies indicated that parental lifestyle and paternal environmental exposures (e.g. stress, nutritional status, smoking) can change the metabolic health and disease susceptibility in offspring, which is known as the hypothesis of Developmental Origins of Health and Disease (DOHaD) [6, 7]. Most of these studies focused on (epigenetic) changes in offspring induced by maternal exposures before, during or after pregnancy [8]. However, an increasing number of studies in rodents confirm that the offspring's epigenetic profile may also be influenced by paternal preconceptional exposures [9, 10, 11]. For example, a study by Ng et al. [12] observed beta‐cell dysfunction in rat F1 female offspring, whose fathers were fed a preconceptional high fat diet. Furthermore, Carone et al. [13] fed paternal mice a preconceptional low protein diet (LPD), resulting in an overall decreased expression of *Pparα* (a key transcription factor involved in mitochondrial metabolism) in offspring. Paternal exposure to benzo[a]pyrene, a well‐known cigarette smoke‐derived genotoxic compound, also affected the male offsprings’ metabolic health by reducing mitochondrial DNA copy number, mitochondrial activity and oxidative stress induced DNA damage [14]. A study in humans confirmed that paternal preconceptional smoking can indeed alter the levels of oxidative DNA damage in their offspring at birth [15]. Altogether, these results indicate that paternal preconception environmental/lifestyle exposures can have intergenerational effects on the metabolic health of offspring, also in apparently healthy subjects, which could further explain inter‐individual variations.

The aim of this study was therefore to elucidate whether the paternal nutritional status has intergenerational effects on oxidative stress‐related DNA damage and indices of mitochondrial metabolic dysfunction in the livers and lungs of male offspring. Liver and lung were selected as organs in which accumulation of DNA damage can have long‐term health consequences, including cancer, providing a relevant context to study inter‐individual variation in oxidative stress–induced genomic instability. Male C57BL/6 mice received either a control diet or an LPD for 7 weeks (from 3 to 10 weeks of age) and were mated with female mice reared on a control diet. In order to determine the potential intergenerational effects of the father's nutritional status on DNA damage in offspring, 8‐oxo‐dG levels were assessed. Additionally, mitochondrial DNA copy number, mitochondrial activity, and the expression of key genes involved in mitochondrial biogenesis and antioxidant defense were assessed.

## 2 Experimental Section

### 2.1 Animal Breeding and Diets

**FIGURE 1:**
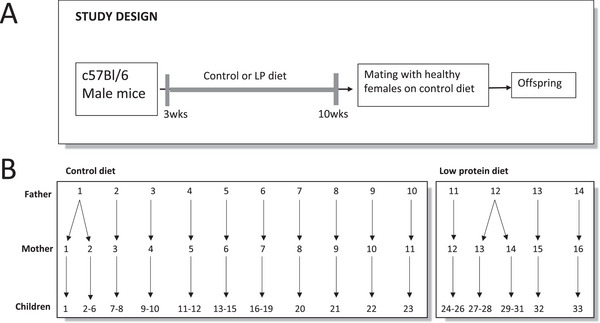
Overview of experimental design. (A) Fathers received a control diet or low protein (LP) diet from 3 to 10 weeks of age. Females were raised on a control diet, which was continued from pregnancy until weaning. All offspring received control diets throughout the study. (B) number of animals involved in the breeding scheme. 10 Fathers received control diet and 4 received a low protein diet. These animals were subsequently mated with 11 and 5 female mice, resulting in 23 control offspring mice and 10 offspring mice whose father received a low protein diet.

Male C57BL/6 mice were fed AIN‐93G based diets ad libitum, containing either 18.8% protein (control) or 9.5% kcal from protein (LPD), starting three weeks after birth (see Supporting Information S1 for details regarding nutrient compositions of the different diets). The animals were mated at ten weeks of age with female mice reared on a control diet (18.8% protein). The male mice were kept in the female cages for three days. Female mice received the control diet throughout the breeding period, pregnancy (21 days) and after giving birth. After weaning, all offspring were weighed and received a control diet throughout the study. Unfortunately, tissues of female offspring were not available, because these animals were used for another experiment [11]. Additionally, sex differences are known to exist [16]. Therefore, only male offspring were investigated, allowing adherence to the 3R principles by maximizing the use of existing animals and avoiding the need for additional experiments. To avoid postnatal litter effects, pups were cross‐fostered by exposure group 2 days after dams gave birth. Pups from 2 to 3 dams were pooled and housed in a litter of 8–10 pups per nursing dam. Male and female pups were housed together until weaning. Cross‐fostered pups were weaned on postnatal day 21, housed by sex to avoid mating, and were fed the control diet throughout the rest of the experiment. Approximately five males by group were housed together in one cage. The bodyweight of pups was recorded at birth and at the end of the experiment. The male progenies were sacrificed at 9 months of age (*n* = 23 for the control group from 10 different fathers and *n* = 10 for the LPD group from 4 different fathers), after which liver and lung tissues were extracted, snap‐frozen in liquid nitrogen and stored at −80°C. The experimental design is visualized in Figure 1. All animal procedures were approved by the Georgetown University Animal Care and Use Committee (protocol #2016‐1172), and the experiments were performed following the National Institutes of Health guidelines for the proper and humane use of animals in biomedical research [11].

### 2.2 DNA and RNA Isolation

Liver and lung tissues were crushed in liquid nitrogen, after which DNA and RNA was isolated, using respectively a standard phenol‐chloroform extraction method or a RNeasy Minikit^plus^ (Qiagen, Valencia, CA) according to the manufacturer's instructions. DNA isolation was optimized for analysis of 8‐oxo‐dG to minimize artificial induction of oxidated DNA bases by using radical‐free phenol, minimizing exposure to oxygen, and adding 1 mM of the metal chelator deferoxamine mesylate and 20 mM 2,2,6,6‐tetramethylpiperidine‐N‐oxyl. An additional DNAse digestion step was included in the RNA isolation procedure using gDNA Eliminator spin columns. The purity and concentrations of the samples was assessed using a Nanodrop‐1000 spectrophotometer (ThermoFisher). Finally, the isolated DNA and RNA samples were stored at −20°C and −80°C, respectively, for further analysis.

### 2.3 Oxidatively Induced DNA Damage

The oxidative stress induced DNA damage was measured by assessing the levels of 8‐oxo‐dG adducts relative to normal dG [17]. Thirty µg of DNA from liver and lung samples were digested in duplicate by the addition of 6 µL NaAc (0.5 M), 9 µL ZnC1_2_ (10 mM) and 1.5 µL nuclease P1 (1 U/µL), following incubation for 90 min at 37°C. Subsequently, 30 µL Tris (0.5 M, pH 7.4), and 1.5 µL alkaline phosphatase (1 U/µL) was added for 45 min at 37°C. The 8‐oxo‐dG adducts were assessed using a SupelCosil LC‐18‐S column with an electrochemical detector (DECADE II, ANTEC). The mobile phase (15 mM KH_2_PO_4_, 2 mM K_2_HPO_4_, 4 mM KCl and 0.1 mM EDTA, 5% methanol) was used to stabilize the ECD signal, after which the 8‐oxo‐dG levels were determined using standard dilutions (125, 250, 375 and 500 fmol/injection). Finally, the dG‐levels were determined by UV‐absorption at 260 nm, and quantified using standard dilutions (1, 5, 10 nmol/50 µL).

### 2.4 Gene Expression and Mitochondrial Copy Number

Isolated RNA samples were first reverse‐transcribed into cDNA (400 ng), using an iScript cDNA synthesis kit (Bio‐Rad 170‐8891) and thermal cycler (Biometra, Westburg) following the manufacturer's instructions. Efficiency and stability of primers of interest were determined using standard curve dilutions. QPCR was performed for quantitative assessment of gene expression and mitochondrial (mt) DNA copy number with a CFX‐connector and iCycler Thermocycler (Bio‐Rad) using SYBR Green. The cDNA (gene expression analyses), template DNA (mtDNA copy number analyses), and controls (no template, no enzyme and blanc) were diluted 1/50 and added (5 µL) to a 96‐well plate and analysed in triplicate. Subsequently, the mastermix, consisting out of 1.2 µL primer pair (10 µM), 10 µL SYBR Green and 3.8 µL nuclease‐free H_2_O, was added, in aliquots of 20 µL. All genes analyzed and their corresponding primers are presented in Table 1. QPCR was performed in concordance to a 2‐step‐AMP protocol with melting curve. QPCR started with denaturation at 95°C for 3 min., followed by 45 cycles at 58°C (45 sec) as annealing temperature and a final extension at 55°C for 1 min. The specificity of amplification and absence of primer dimers was confirmed using the melting curve analysis (MyiQ Software system, Bio‐Rad). Data was expressed as Ct values of genes of interest, relative to the endogenous reference housekeeping gene *Ribosomal Protein L13α (Rpl13α)* (fold change), using the 2^−ΔΔCt^ method.

**TABLE 1:** Overview of primers (forward (F), reverse (R)) used in qPCR experiments for gene expression.

Gene	5’‐Primer (F)	3’‐Primer (R)	UniProt identifier
*Ndufb3*	GGACGCCATTAGAAACGGTGCA	ACACTCGGGAAGGTGATGTTGC	Q9CQZ6
*Pgc1α*	GAATCAAGCCACTACAGACACCG	CATCCCTCTTGAGCCTTTCGTG	O70343
*PParα*	ACCACTACGGAGTTCACGCATG	GAATCTTGCAGCTCCGATCACAC	P23204
*Tfam*	GAGGCAAAGGATGATTCGGCTC	CGAATCCTATCATCTTTAGCAAGC	P40630
*Nqo1*	GCCGAACACAAGAAGCTGGAAG	GGCAAATCCTGCTACGAGCACT	Q64669
*γ‐Gcs*	ACACCTGGATGATGCCAACGAG	CCTCCATTGGTCGGAACTCTAC	P97494
*Cat*	CGGCACATGAATGGCTATGGATC	AAGCCTTCCTGCCTCTCCAACA	P24270
*Rpl13α*	CTGCTCTCAAGGTTGTTCGGCT	CCTTCCGTTTCTCCTCCAGAGT	P19253

**Abbreviations;** Cat, catalase;Ndufb3, NADH: ubiquinone oxidoreductase subunit b3; Nqo1, NAD(P)H:quinone oxidoreductase‐1; Pgc1α, PPARγ coactivator‐1a; PParα, peroxisome proliferator‐activated receptor α; Rpl13α, Ribosomal Protein L13α; Tfam, mitochondrial transcription factor A; γ‐Gcs, γ‐ glutamylcysteine synthetase.

### 2.5 Citrate Synthase Activity Assay

Liver and lung tissue (5% wt/vol) was homogenized in SET buffer (250 mM sucrose, 2 mM EDTA, 10 mM Tris, pH 7.4) using a 20‐gauge needle (0.9 mm diameter) fitted to an RNase‐free syringe. Samples were subsequently centrifuged (10 min, 14.000 g, 4°C), after which the supernatant was isolated and used for the CS activity assay [18]. Activity was measured spectrophotometrically in duplicate (300 rpm shake, 412 nm, 30s intervals, 50 readings, 37°C) and finally corrected for total protein content. Total protein content was determined using a specific BCA protein assay kit (ThermoFisher Scientific, 23225) according to manufacturer's instructions.

### 2.6 Statistics

Data are presented as the average and standard error of the mean (SEM). Since the dietary intervention was applied to fathers, the father should be considered as the experimental unit. Although the study comprised 23 control and 10 experimental animals in total, the sample size for individual analyses varied because not all assays could be performed in all animals; corresponding sample sizes are indicated in the figure legends. Data of individual offspring were analyzed using linear mixed‐effects models in SPSS, with paternal diet (control vs low protein) as a fixed effect and father identity included as a random effect to account for non‐independence of pups from the same litter. *P*<0.05 was considered statistically significant. Power calculations based on the study design indicated that this study had 80% power to detect a difference of approximately 1.14 standard deviations (power calculations are provided in Supplementary Information S2). Although the experimental design was unbalanced, statistical power was maintained by the inclusion of a larger number of control animals, thereby ensuring sufficient robustness for detecting biologically relevant effects.

## 3 Results

### 3.1 Influence of Paternal Malnutrition on Body Weights

Paternal mice consumed either a control diet or LPD from 3 to 10 weeks of age. Compared to control, there was no difference in sex‐distribution of litters and LPD father's bodyweight of (data were reported in reference 11). We examined the male offspring resulting from mating between control and LPD male mice with female mice reared on a control diet. No differences in birth weight (1.7 ± 0.1 g and 1.6 ± 0.1 g for litter of control fathers and fathers that consumed an LPD, respectively, *p* = 0.45) or weight gain during life were observed. Body weight of the offspring at the end of the experiment (9 months of age) were 42.9 ± 0.4 g and 42.8 ± 1.9 g, for offspring of fathers that received a control diet and an LPD, respectively.

### 3.2 Oxidative Stress Induced DNA Damage

**FIGURE 2:**
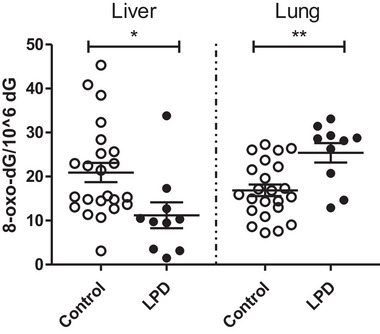
Oxidatively‐induced DNA damage in liver and lung of offspring mice whose fathers were exposed to either a control diet or low protein diet (LPD). Expressed as 8‐oxo‐dG relative to the number of dG molecules. Indicated are mean± SEM from 23 biological replicates of control animals and 10 animals whose father received a low protein diet (LPD), *p<0.05, **p≤0.01 vs control, assessed by linear mixed‐effects models with paternal diet (control vs low protein) as a fixed effect and father identity included as a random effect to account for non‐independence of pups from the same litter.

In order to assess the oxidative stress‐related DNA damage in liver and lung tissue of offspring mice, 8‐oxo‐dG levels relative to dG molecules were determined using HPLC‐ECD (Figure 2). Compared to the paternal control diet, the paternal LPD resulted in a two‐fold decrease in levels of 8‐oxo‐dG/10^6^ dG in offspring's liver (p = 0.03). In contrast, 8‐oxo‐dG/10^6^ dG levels were significantly increased in lungs of offspring whose fathers were exposed to an LPD compared to the paternal control diet (p = 0.01).

### 3.3 Mitochondrial Abundance

**FIGURE 3:**
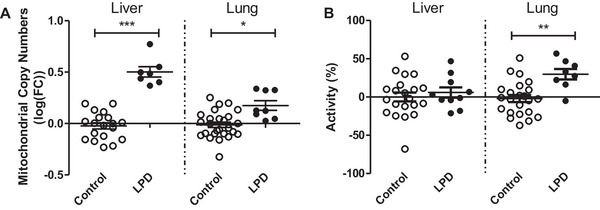
(A) log fold change (FC) of mtDNA copy number in male offspring of fathers exposed to control diet or LPD in both liver and lung tissues. (B) Enzyme activity of citrate synthase in homogenized liver and lung tissue of offspring whose fathers were exposed to a low protein diet, expressed as percentage difference compared to control animals. Indicated are means ±SEM. Mitochondrial copy number (A) were assessed in 19 (liver) and 23 (lung) biological replicates of controls and 7 and 8 biological replicates after paternal LPD for liver and lung, respectively. Citrate synthase activity (B) was assessed in 21 (liver) and 23 (lung) biological replicates of controls and 10 and 8 biological replicates after paternal LPD for liver and lung, respectively. *p<0.05, **p<0.01, ***p<0.001 vs control, assessed by linear mixed‐effects models with paternal diet (control vs low protein) as a fixed effect and father identity included as a random effect to account for non‐independence of pups from the same litter.

Mitochondria are a well‐known source of intracellular oxidative stress [19]. In order to get an indication of differences in the number of mitochondria impacted by the paternal diet, the mtDNA copy number relative to the nuclear DNA was assessed in both liver and lung tissues collected from the offspring (Figure 3A). Compared to the control group, the mtDNA copy number increased by 55% and ∼15% in liver (*p*<0.0001) and in lung (*p* = 0.027) tissues, respectively, of male offspring whose fathers received an LPD diet (Figure 3A).

However, this method may not always be reliable because some conditions can increasing the mitochondrial content, while decreasing the mtDNA copy number [20]. Therefore, a better estimate of the number of mitochondria is through the assessment of the level of activity of citrate synthase (CS), a mitochondrial matrix enzyme of the Krebs cycle. Largely in line with mtDNA copy number data, the activity of CS was higher in both liver and lung tissue of offspring whose fathers received an LPD compared with controls, with a nonsignificant 6% increase in enzyme activity in the liver (*p* = 0.13), and a 22% statistically significant increase in the lungs (*p* = 0.008; Figure 3B).

### 3.4 Mitochondrial Biogenesis

**FIGURE 4:**
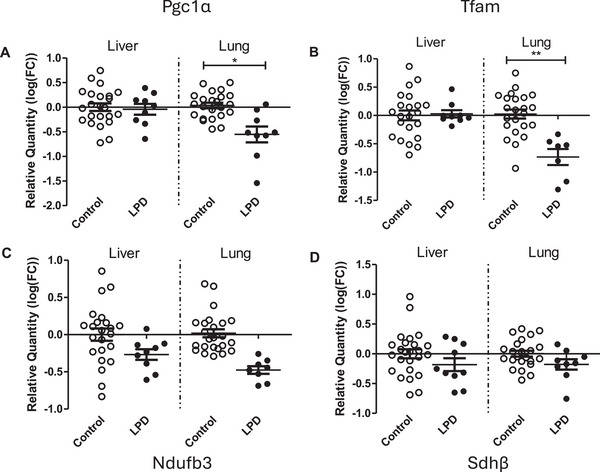
mRNA expression of genes involved in mitochondrial biogenesis and function in liver and lung tissue of offspring whose fathers were exposed to a control or low protein diet. Expression was calculated relative to housekeeping gene Rpl13α (calibrator gene). A. Pgc‐1α B. Tfam C. Ndufb3 D. Sdhβ. Indicated are means and SEM. Pgc1a (A) was assessed in 23 biological replicates of controls and 9 biological replicates after paternal LPD for liver and lung. Tfam (B) was assessed in 22 (liver) and 23 (lung) biological replicates of controls and 9 or 7 biological replicates after paternal LPD for liver and lung, respectively. Ndufb3 (C) was assessed in 23 (liver or lung) biological replicates of controls and 9 or 8 biological replicates after paternal LPD for liver and lung, respectively. Sdhβ (D) was assessed in 23 (liver or lung) biological replicates of controls and 10 or 9 biological replicates after paternal LPD for liver and lung, respectively. *p<0.05, **p<0.01 vs control assessed by linear mixed‐effects models with paternal diet (control vs low protein) as a fixed effect and father identity included as a random effect to account for non‐independence of pups from the same litter.

The abundance of transcriptional co‐activator, *Pgc‐1α*, and transcription factors *Pparα* and *Tfam* were assessed using qPCR, because of their essential role in mitochondrial biogenesis and transcription of the mitochondrial genome. Gene expression of both *Pgc‐1α* and *Tfam* was respectively 56 and 73% lower in lung tissue of offspring whose fathers were exposed to an LPD (*p* = 0.026; *p* = 0.031, respectively, Figure 4A, B). No changes in gene expression were observed in the liver tissue. In addition, expression of 2 sub‐units of electron transport chain complexes were overall highly variable within each tissue (*Ndufb3*: complex II; *sdhβ*: complex II) and did not statistically differ between lungs of paternal LPD offspring, compared to offspring whose fathers received a normal diet (Figures 3C and D, respectively).

### 3.5 Protective Gene Expression

**FIGURE 5:**
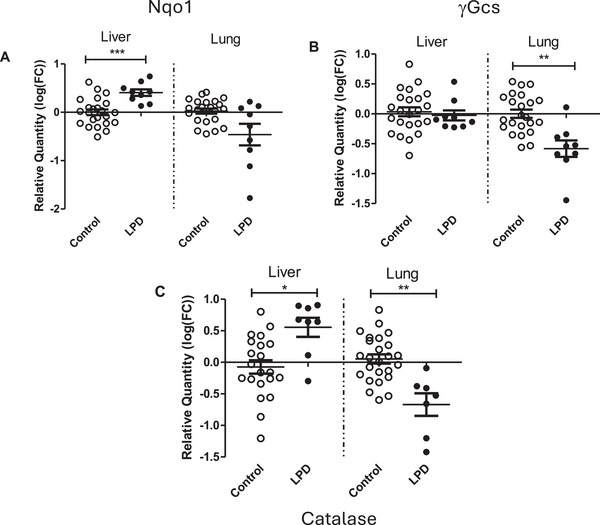
mRNA expression of cytoprotective enzymes in liver and lung tissue, of offspring whose fathers were exposed to a control diet, or LPD, relative to housekeeping gene Rpl13α (calibrator gene). A. Nqo1 B. γ‐Gcs C. Catalase. Data are shown as means and SEM. Nqo1 (A) was assessed in 23 (liver or lung) biological replicates of controls and 9 biological replicates after paternal LPD for liver and lung, respectively. γGcs (B) was assessed in 23 (liver or lung) biological replicates of controls and 9 biological replicates after paternal LPD for liver and lung, respectively. Catalase (C) was assessed in 21 (liver) and 23 (lung) biological replicates of controls and 8 or 7 biological replicates after paternal LPD for liver and lung, respectively. *p<0.05, **p<0.01 vs control assessed by linear mixed‐effects models with paternal diet (control vs low protein) as a fixed effect and father identity included as a random effect to account for non‐independence of pups from the same litter.

One of the most important mechanisms involved in counteracting oxidative stress, is the stimulation of gene expression of various cytoprotective enzymes. The mRNA expression of antioxidant enzymes, *Nqo1, γ‐Gcs* and *Catalase* was assessed using qPCR. The paternal LPD resulted in significantly higher expression of *Nqo1* and *Cat* in liver tissue (*p* = 0.003; *p* = 0.048, Figure 5A, C) when compared to offspring of paternal mice on a control diet. In contrast in lung tissue, gene expression of *γ‐Gcs* and *Catalase* were lower in LPD offspring when compared to controls (*p* = 0.034; *p* = 0.01, respectively).

## 4 Discussion

Levels of DNA damage in human studies show large inter‐individual variations, which cannot be fully explained by differences in exposure to DNA damaging compounds, nor by genetic variations in relevant genes, such as genes in metabolism and DNA repair [21]. Thus, other sources for this level of variation need to be explored. Extensive research has been conducted on associations between periconceptional maternal diet and effects on offspring's health, but the influence of the father's diet is emerging [11, 14, 22, 23, 24, 25]. We previously showed that preconceptional paternal smoking behavior can affect DNA damage in lymphocytes of newborns [15]. We additionally observed that paternal exposure to cigarette smoke constituent benzo[a]pyrene can affect mitochondrial activity and the expression of DNA repair genes [14]. Previous research found effects of LPD in offspring health outcomes. For example, a study done by Watkins et al. [26] and our previous study [11] found changes in the AMPK pathway, which is important for cellular energy homeostasis and mitochondrial function. Here, we investigated whether paternal malnutrition, more specifically LPD, can affect levels of DNA damage in offspring, mitochondrion content and levels of intracellular oxidative stress and subsequent levels of oxidative DNA damage in offspring.

Paternal LPD resulted in lower 8‐oxo‐dG levels in the liver, but simultaneously higher levels of 8‐oxo‐dG were found in lung tissue of the same male offspring. These findings are consistent with previous studies that indicate that the paternal nutritional status has an organ‐specific influence on the offspring. For instance, Day et al. [27] suggested organ‐specific effects through transgenerational influences induced by paternal alcohol consumption. Although this organ discrepancy cannot be explained yet, the development of the lung (emerges at days 9‐9.5) compared to liver (days 3‐3.5) depends on different transcriptional factors, which may be differentially epigenetically affected by an LPD [28]. However, more studies are needed to understand how paternal diet may affect the development of various organs and the development of oxidant/antioxidant balances and which sperm‐related factors are involved.

Mitochondria are known as a source of intracellular ROS [19]. These organelles are maternally inherited, but we previously showed that paternal lifestyle and exposures can still affect mitochondrial numbers and function in offspring [14]. When ROS production in mitochondria is persistently elevated, and the scavenging by antioxidants is not adequate, not only the mtDNA, but also the nuclear DNA may come under oxidative attack. Therefore, we hypothesized that the altered levels of 8‐oxo‐dG originated from a modified balance between ROS formation and scavenging, both in the offspring's liver and lung tissue. This idea is in line with the observed increase in mitochondrial abundance in liver and lung, determined by the mtDNA copy number as well as the level of activity of CS. Although the mtDNA copy number was increased in the offspring's liver, whose father received the LPD, the CS activity was not significantly increased. Since the CS activity assessment is considered to be a more reliable marker for mitochondrial abundance [20], it can therefore be concluded that the paternal LPD likely did not result in drastic differences in mitochondrial abundance in the liver. In lung tissue, however, both the mtDNA copy number and CS activity were significantly higher in offspring whose fathers received an LPD, which potentially may have contributed to higher levels of 8‐oxo‐dG.

Mitochondrial biogenesis is a key factor in determining mitochondrial abundance [29]. PGC‐1α is the master regulator of mitochondrial biogenesis through the functional interaction with various transcription factors, such as Tfam. Tfam is a main determinant of mtDNA copy number, due to its role in transcription and replication initiation of the mitochondrial genome [30]. Surprisingly, despite the higher levels of mitochondrial copy number and CS activity, mRNA expression of *Pgc‐1α* and *Tfam* was lower in lung tissue of offspring whose fathers were exposed to LPD. This contradiction could possibly be explained by a discrepancy between gene expression and subsequent protein levels. Indeed, a study by Aoi et al. [31] reported that expression of *Pgc‐1α* was regulated by miRNAs upon environmental stimuli. Since miRNA can inhibit protein synthesis by blocking translation, it would be interesting to study miRNA abundances in offspring's tissues and paternal sperm in future studies. Another explanation could be that the increased mitochondrial abundance resulted in a negative feedback loop, which subsequently led to the downregulation of the master regulator *Pgc‐1α* and its downstream targets *(Tfam)* in lung. Interestingly, Baldelli et al. [32] reported that Pgc‐1α regulates factor Nrf2, in response to ROS. Nrf2 subsequently upregulates cytoprotective genes [33], including various antioxidant genes. Indeed, we found that a paternal LPD increased the mRNA expression of the protective enzymes *Nqo1* and *Cat* in the liver. In lung tissue, however, the mRNA expression of *γ‐Gcs* and *Cat* was lower. This may explain the altered scavenging capacity of ROS noticeable as altered 8‐oxo‐dG levels.

A limitation of the current study is its descriptive nature, with the underlying mechanisms still needing further attention. However, literature shows that sperm indeed contains potential programming factors that influence the offspring biology. These factors include small noncoding RNAs (sncRNA), such as miRNAs and nuclear and mitochondrial‐derived tRNA and rRNA fragments [34]. Importantly, sperm sncRNA can be modulated by paternal lifestyle/ exposure [35]. These sperm related factors may be used by the fetus as programming cues to adapt to conditions in utero, but different conditions may be met after birth. In this study, adapting to low protein conditions, but encountering nutrient‐rich postnatal diets, changed oxidative stress in a tissue specific manner. Previous studies on maternal LPD during pregnancy showed that oxidative stress can indeed be induced by limiting glutathione synthesis and impairing antioxidant defenses [36]. We showed here that paternal preconceptional exposure to a LPD may have similar effects in offspring. While paternal LPD has been shown to perturb offspring metabolic health, gene expression and epigenetic regulation [37, 38], direct evidence for oxidative stress induced DNA damage in the offspring has so far been limited. Oxidative stress induced DNA damage in sperm has been proposed to act as a molecular cue during fertilization [15], influencing early embryonic development and potentially initiating programming effects that persist into later life. Notably, McPherson et al. [39] demonstrated that fortification of the paternal diet by antioxidants to mitigate sperm DNA damage also reversed metabolic disorders in offspring, suggesting that nutritional intervention in fathers may offer a strategy to prevent adverse effects in offspring. Recognizing mismatched diets and conditions in utero and after birth, emphasizes the importance of promoting parental lifestyles to improve future generations' health outcomes. More specifically, this study showed that paternal malnutrition can influence mitochondrial function and the oxidative stress balance leading to variation in DNA damage. The large variation in oxidative DNA damage as observed in population studies may thus at least partly find its origin in parental preconceptional diets and lifestyle. Our study adds to the growing literature showing that the progeny's health in adulthood is linked to periconception parental exposures. More attention to both maternal and paternal periconceptional health could prevent diseases in offspring normally associated with growing old.

## Conflicts of Interest

The authors declare no conflicts of interest.

## Supporting information




**Supporting File 1**: mnfr70470‐sup‐0001‐TableS1.docx.

**Supporting File 2**: mnfr70470‐sup‐0002‐SuppMat.docx.

## Data Availability

The data that support the findings of this study are available from the corresponding author upon reasonable request.
