# Supplementary material for: Paternal Malnutrition has Organ‐Specific Intergenerational Effects on Mitochondrial Function and Oxidative Stress Induced DNA Damage in Male Mouse Offspring
Source: Mol Nutr Food Res. 2026 Apr 17;70:e70470. doi: 10.1002/mnfr.70470 (PMC13088000; doi:10.1002/mnfr.70470)
Supplement: Supplementary file 1 — Supporting File 1: mnfr70470‐sup‐0001‐TableS1.docx. [file MNFR-70-e70470-s002.docx]

**Table S1.** Composition of experimental diets

| *Ingredients* | *g/kg* | |
| --- | --- | --- |
|  | **Control (CO)** | **Low Protein (LP)** |
| Casein | 200.000 | 100.000 |
| L-Cystine | 3.000 | 2.000 |
| Corn Starch | 397.386 | 496.586 |
| Maltodextrin | 132.000 | 132.000 |
| Sucrose | 100.000 | 108.000 |
| Corn Oil | 60.000 | 60.000 |
| Soybean Oil | 10.000 | 10.000 |
| Cellulose | 50.000 | 50.000 |
| Vitamin Mix | 10.000 | 10.000 |
|  |  |  |
| **Protein (%)** | 17.7 | 8.9 |
| **Carbohydrate (%)** | 60.1 | 69.0 |
| **Fat (%)** | 7.2 | 7.1 |
| **Kcal/g** | **3.8** | **3.8** |

Control (TD.08819) and LP (TD.130983) are AIN93G-based diets containing either 17.7%

or 8.9% energy from protein. Diets were manufactured by Envigo Teklad Diets.
