# Supplementary material for: Paternal Malnutrition has Organ‐Specific Intergenerational Effects on Mitochondrial Function and Oxidative Stress Induced DNA Damage in Male Mouse Offspring
Source: Mol Nutr Food Res. 2026 Apr 17;70:e70470. doi: 10.1002/mnfr.70470 (PMC13088000; doi:10.1002/mnfr.70470)
Supplement: Supplementary file 2 — Supporting File 2: mnfr70470‐sup‐0002‐SuppMat.docx. [file MNFR-70-e70470-s001.docx]

**Supplementary file 2**

**Power analysis**

**S**tatistical power was assessed using three complementary approaches.

1.

We performed an *a-priori* power analysis based on variance components obtained from an earlier dataset (see Figure S2 in this supplement). We used a two-level hierarchical approximation in which offspring were nested within mothers and mothers within fathers. For each father $i$with mothers $j$, offspring counts $n_{ij}$, and total offspring $N_{i}$, the sampling variance of the father–level mean was:

$$\mathrm{Var}(Y_{i})=\sigma_{F}^{2}+\sigma_{M}^{2}\frac{\sum_{j} n_{ij}^{2}}{N_{i}^{2}}+\frac{\sigma_{R}^{2}}{N_{i}}\text{ ⁣},$$

where $\sigma_{F}^{2}$, $\sigma_{M}^{2}$, and $\sigma_{R}^{2}$ denote paternal, maternal-within-father, and residual variance components, respectively. Group-mean variances were obtained by summing these father-level variances and dividing by the squared number of fathers per group. Power for detecting a group difference $\delta$was computed as:

$\text{Power}=\Phi\text{ ⁣}\left( \frac{\delta}{SE_{D}} - 1.96 \right),$ with two-sided $\alpha=0.05$.

In this analysis all fathers were retained even when they contributed only a single offspring, because including all sire clusters reduces sampling variance of the group mean and therefore maximizes statistical power despite the unbalanced design.

2.

After the study was completed, we repeated the calculations using the control-group data and structure.

3.

We conducted a final *post-hoc* power analysis using the variance components obtained from the fitted linear mixed model, in which exposure was included as a fixed factor and mother was nested within father. The full unbalanced dataset (controls and exposed combined) was used to compute father-level means and group-level sampling variances.

Across all approaches, power increased with effect size and with the number of fathers that contributed to the offspring. The final model-based analysis, which reflects the realized variance structure of the experiment, indicates moderate power for detecting effects around 1 SD and high power for larger effects. 80% power was reached for differences of 1.14x SD.

**Table S2. Statistical power for detecting paternal diet effects in the planned vs. observed study**

| Effect Size | Prospective Analysis  σ²ₘ(f)=53.3  σ²ᵣ=32.8 | Ad-hoc Control-Only Analysis  σ²ₘ(f)=15.6  σ²ᵣ=30.5 | Final full Mixed-Model Analysis  σ²ₘ(f)=9.4  σ²ᵣ=23.2 |
| --- | --- | --- | --- |
| 0.5 SD | 0.152 | 0.163 | 0.233 |
| 1.0 SD | 0.463 | 0.498 | 0.690 |
| 1.5 SD | 0.800 | 0.835 | 0.958 |
| 2.0 SD | 0.962 | 0.975 | 0.998 |

The a-priori power analysis was based on variance components obtained from an earlier dataset in which 17 unexposed sires were mated with 1 or 2 different dams, and subsequently 2 offspring animals of each nest were analyzed for the level of 8-oxodG in lung DNA. This resulted in the experimental setup with 17 fathers, 22 mothers and 44 offspring animals (see figure below). A mixed linear model was applied to determine the variance coming from the father $(\sigma_{F)}^{2}$, the mother (nested within the fathers, $\sigma_{M}^{2}$) and the residual variance ($\sigma_{R}^{2})$. This variance was used for power calculations as described above.


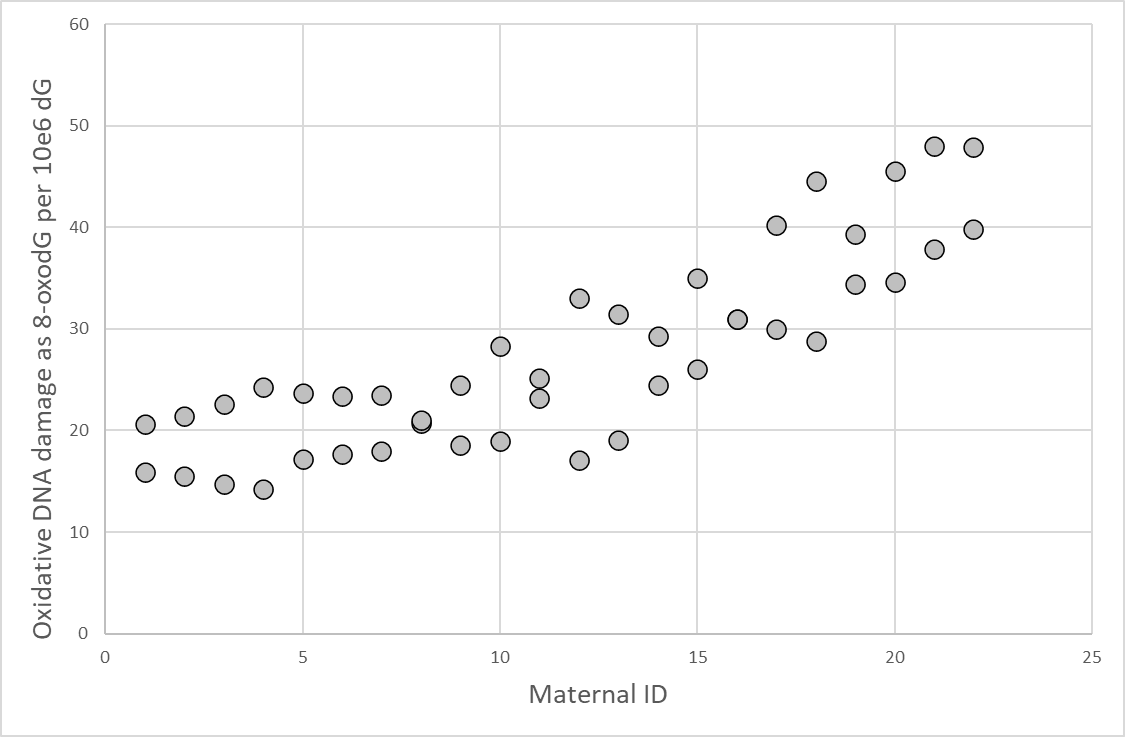

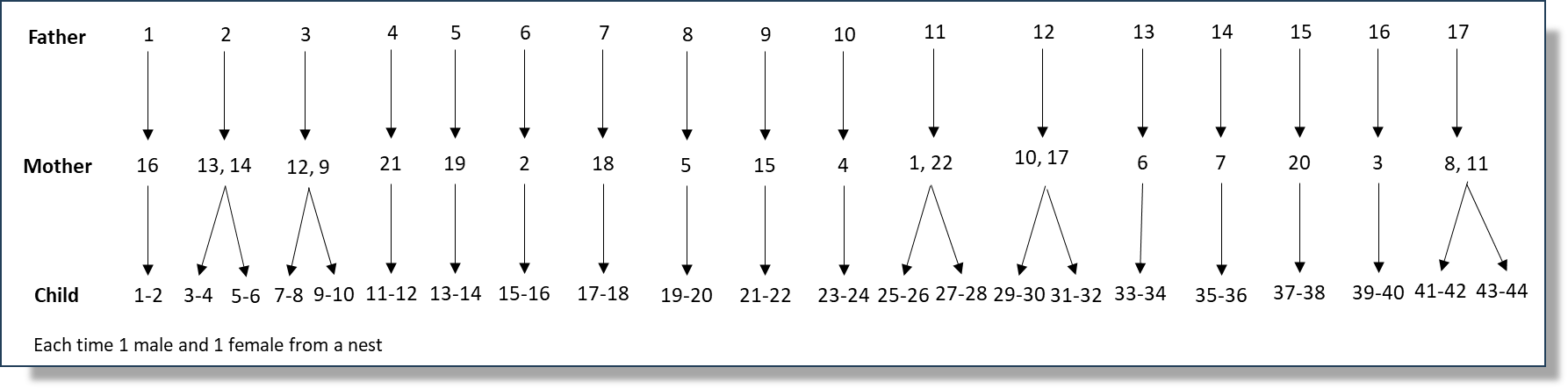


**Figure S2**. *DNA damage was assessed in two offspring per nest, and nests were ordered by their mean DNA damage levels. Consistent with the nested design, sibling offspring exhibited more similar DNA damage levels compared with offspring from different nests.*
